# [Retracted] Protective effects of baicalin on rabbit articular chondrocytes *in vitro*

**DOI:** 10.3892/etm.2023.12236

**Published:** 2023-10-02

**Authors:** Xianyuan Huang, Huayu Wu, Liqin Wang, Li Zheng, Jinmin Zhao

Exp Ther Med 13:1267–1274, 2017; DOI: 10.3892/etm.2017.4116

Following the publication of the above paper, and also a corrigendum in 2017 (doi: 10.3892/etm.2021.9768) that was intended to rectify the issue of duplicated data panels featured in Figs. 3 and 5, it has been drawn to the Editor’s attention by a concerned reader that there was a further data duplication event comparing the panels in the original version of Fig. 3 that went undetected, albeit that the identical areas exhibited different intensities of fluorescence. In addition, for the immunohistochemical staining images shown in Fig. 8 on p. 1273, the ‘Day 6 / 2.5 μmol/l’ data panel appeared to include an anomalous overlap of matching data within the data panel itself. Owing to the fact that this paper has already had a published corrigendum and given the new information that has come to light, the Editor of *Experimental and Therapeutic Medicine* has decided that it should be retracted from the Journal on account of a lack of confidence in the originally presented data. The authors were asked for an explanation to account for these concerns, but the Editorial Office did not receive a satisfactory reply. The Editor apologizes to the readership for any inconvenience caused.

